# Identification of a Kinase Profile that Predicts Chromosome Damage Induced by Small Molecule Kinase Inhibitors

**DOI:** 10.1371/journal.pcbi.1000446

**Published:** 2009-07-24

**Authors:** Andrew J. Olaharski, Nina Gonzaludo, Hans Bitter, David Goldstein, Stephan Kirchner, Hirdesh Uppal, Kyle Kolaja

**Affiliations:** 1 Non Clinical Safety, Roche Palo Alto LLC, Palo Alto, California, United States of America; 2 Pharma Research Scientific Informatics, Roche Palo Alto LLC, Palo Alto, California, United States of America; 3 Medicinal Chemistry, Roche Palo Alto LLC, Palo Alto, California, United States of America; 4 Non Clinical Safety, F. Hoffman-La Roche AG, Basel, Switzerland; University of Houston, United States of America

## Abstract

Kinases are heavily pursued pharmaceutical targets because of their mechanistic role in many diseases. Small molecule kinase inhibitors (SMKIs) are a compound class that includes marketed drugs and compounds in various stages of drug development. While effective, many SMKIs have been associated with toxicity including chromosomal damage. Screening for kinase-mediated toxicity as early as possible is crucial, as is a better understanding of how off-target kinase inhibition may give rise to chromosomal damage. To that end, we employed a competitive binding assay and an analytical method to predict the toxicity of SMKIs. Specifically, we developed a model based on the binding affinity of SMKIs to a panel of kinases to predict whether a compound tests positive for chromosome damage. As training data, we used the binding affinity of 113 SMKIs against a representative subset of all kinases (290 kinases), yielding a 113×290 data matrix. Additionally, these 113 SMKIs were tested for genotoxicity in an in vitro micronucleus test (MNT). Among a variety of models from our analytical toolbox, we selected using cross-validation a combination of feature selection and pattern recognition techniques: Kolmogorov-Smirnov/T-test hybrid as a univariate filter, followed by Random Forests for feature selection and Support Vector Machines (SVM) for pattern recognition. Feature selection identified 21 kinases predictive of MNT. Using the corresponding binding affinities, the SVM could accurately predict MNT results with 85% accuracy (68% sensitivity, 91% specificity). This indicates that kinase inhibition profiles are predictive of SMKI genotoxicity. While in vitro testing is required for regulatory review, our analysis identified a fast and cost-efficient method for screening out compounds earlier in drug development. Equally important, by identifying a panel of kinases predictive of genotoxicity, we provide medicinal chemists a set of kinases to avoid when designing compounds, thereby providing a basis for rational drug design away from genotoxicity.

## Author Summary

Small molecule kinase inhibitors (SMKIs) are a class of chemicals that have successfully been used for the treatment of a number of oncological diseases that are now being pursued by the pharmaceutical industry for inflammatory diseases, such as rheumatoid arthritis. SMKIs are generally designed to specifically inhibit one kinase, but this is challenging due to the structural similarity of the ATP binding pocket amongst different members of the kinase family. The inability to selectively inhibit just one kinase can be problematic, as kinases play key roles in a number of cellular processes. Thus the unwanted inhibition of additional kinases can lead to undesirable toxicities that may halt drug development. One type of toxicity often observed with this class of compounds is damage to chromosomes, which can occur when kinases involved with cell cycle progression or chromosome dynamics are inhibited. Here we demonstrate that mathematical modeling can be used to identify kinases that correlate with chromosome damage, information which can assist medicinal chemists in avoiding certain kinases when synthesizing new chemicals. Generation of this type of information is one of the first steps in beginning to reduce toxicity-based attrition for this class of compounds.

## Introduction

Toxicity is a major cause of attrition in drug development. While identifying liabilities and potential toxicity is difficult and costly, safety issues can become markedly more complex when kinases are the pharmaceutical target. Kinases regulate many basic functions in normal cells. When their activity is altered, kinases can be the mechanistic reason for a cell to acquire an abnormal phenotype. In metabolic, oncologic, viral, cardiovascular and inflammatory diseases, over 150 different kinases, of the over 500 known protein kinase family members, are considered putative drug targets [1]. Marketed small molecule kinase inhibitors (SMKIs) have suitably demonstrated the effectiveness of this therapeutic approach for oncologic indications [2]. SMKIs intended for non-oncologic diseases, however, are increasingly represented in various stages of preclinical and clinical development [1]. Most SMKIs exert their pharmacologic effect by interacting with the ATP binding pocket [3], inhibiting the ability of the kinase to phosphorylate the intended substrate, and blocking downstream signal transduction. Because of the evolutionarily conserved nature of the ATP binding pocket, a SMKI intended to inhibit a particular kinase may potently inhibit dozens of other kinase members across the human kinome [4]. Off-target kinases can be a potential safety liability of this therapeutic class and hinder drug development. The mechanisms by which different toxicities arise as a result of off-target inhibition are not well characterized. Sutent, a highly non-selective inhibitor of multiple tyrosine kinases and Gleevec, a relatively selective Bcr-Abl inhibitor, both increase the risk of cardiotoxicty [5]–[7], though additional, less publicized toxicities, are also common for SMKIs.

Kinases are key regulators of mitosis, as they are intricately involved with precise signaling and the coordination needed for proper replication and segregation of chromosomes into daughter cells [8]–[10]. While kinases may be targeted for their role in pathways associated with a disease of interest, inhibition of kinases may also disrupt normal cellular processes. A frequently observed toxicity for SMKIs is a positive result for chromosomal damage in an assay of DNA integrity, which likely occurs as the result of inhibiting kinases involved in mitosis or chromosomal segregation. The micronucleus test (MNT) is widely regarded as a sensitive assay for genetic toxicity as it is a means to detect either pieces and/or whole chromosomes that appear as a micronucleus in the daughter cell following chemical exposure [11],[12]. A positive result in this assay can hamper or halt drug development, as it is a biomarker of chromosomal damage, which is a hallmark of cancer [13]–[15]. Thus, human exposure to aneugens or clastogens should be attenuated, or avoided altogether, when possible. A small number of kinases, such as the polo-like and aurora kinases [16]–[19], are known to associate with chromosomal damage, however the genotoxic potential associated with inhibiting the majority of the kinome is largely unknown.

MNT results can be considered a surrogate, and sometimes predictive endpoint for carcinogenicity. This study models this endpoint because of its correlation with genotoxicity and the availability of a set of training compounds that have been screened with this assay. Although regulatory agencies require such an *in vitro* assay prior to moving forward with preclinical development, there are advantages to modeling this assay *in silico*. Namely, because of the low-throughput nature of the assay, the drug discovery process would benefit from a cheaper, faster screen that could assist in reducing the number of leads that typically fail at later stages, as well as help design compounds with fewer safety liabilities. Since all promising SMKIs at Roche are tested in kinase inhibition assays, these data present the opportunity to explore possible correlations between SMKI kinase selectivity and the potential to cause chromosomal damage.

The objective of this study was to identify kinases that correlate with chromosomal damage when inhibited. At Roche, we aimed to use these findings as a set of kinases that medicinal chemists should avoid when designing compounds, so as to avoid positive MNT results, thereby reducing attrition rates. By using machine learning methods on data that were already available from early kinase-based high-throughput screens, we were able to identify such a set of kinases and develop a fast and efficient model for predicting whether a compound will test positive for genotoxicity. Besides its novel utility in the drug discovery pipeline, the model also sheds light on the biological mechanisms of genotoxicity, and allows us to create hypotheses for further studies.

## Results

### Dataset

**Figure 1:**
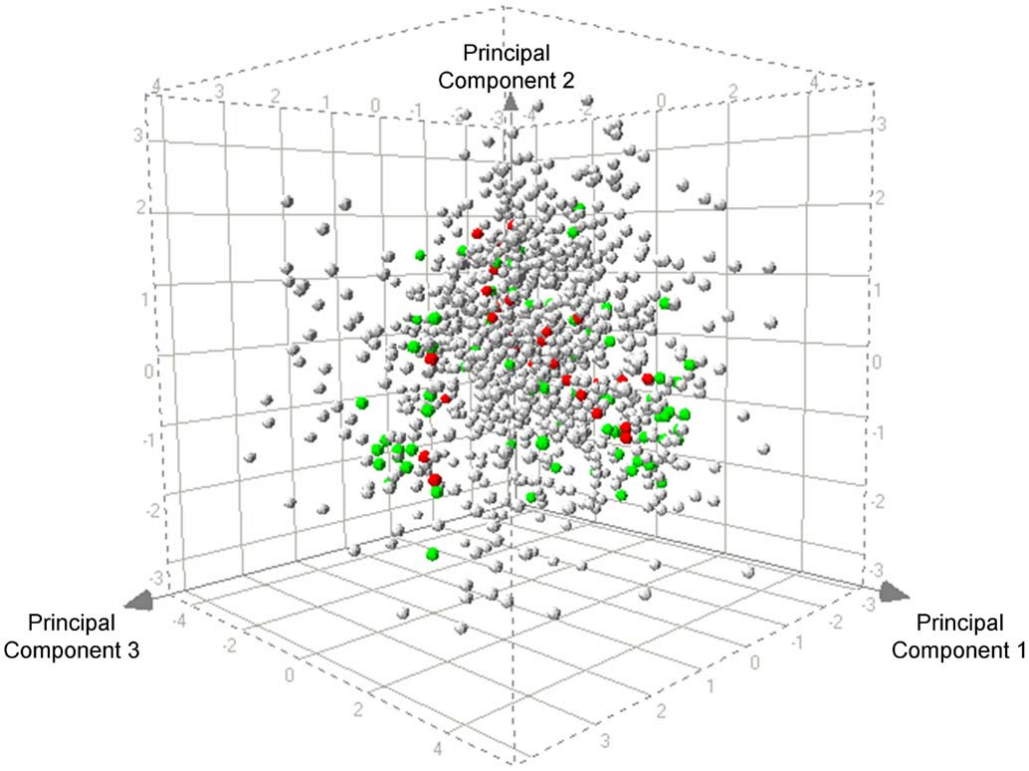
Principal component analysis (PCA) assessing the structural diversity of the 113 SMKIs. Positive and negative micronucleus results are marked in red and green, respectively. Roche SMKIs that have not been tested in the MNT are colored light grey. Compounds selected for this study cover a broad range of chemical space within the Roche SMKIs and reduce redundancy within the test set.

The 113 SMKIs were chosen to represent a diversity of compound properties and structural moieties. Figure 1 shows a Principal Components Analysis (PCA) plot of the training compounds, in color, overlaid on top of a plot of all Roche compounds that have been screened with Ambit Biosciences (San Diego, CA) KINOMEscan assay of 317 kinases. The PCA was based on structural fingerprints, a representation of the molecular structure of each compound. This method of analysis is a means for reducing dimensionality to best explain variability in the data. Figure 1 shows that structures of the 113 compounds are highly variable and sample the chemical space of the entire Roche SMKI library well. With a diverse training set, chances of redundancy are reduced and the model is likely to be more robust to future predictions.

During dataset preprocessing, 27 mutant kinases were removed from the initial panel of 317 kinases as their inclusion and potential selection would be difficult to interpret from biological and mechanistic standpoints. This yielded a panel of 290 kinases, identified in Table S1. An additional 5 uninformative kinases were removed from the panel as their percent inhibition values did not vary significantly across positive and negative SMKIs. Thus, preprocessing yielded a data matrix of 113×285 for machine learning analysis. A heatmap of the full dataset prior to preprocessing can be found in Figure S1. Of the 113 compounds, 30 and 83 SMKIs were classified as MNT positive and negative, respectively.

### Model methods

In the first phase of the analysis, several models were generated, each based on 10×5-fold cross validation for a particular combination of feature selection methods and a binary classifier. In this phase, the best performing feature selection methods were a Kolmogorov-Smirnov/T-test hybrid algorithm, followed by Random Forests. The most informative features were then input into a non-linear Support Vector Machines (SVM) classifier.

The Kolmogorov-Smirnov/T-test algorithm is a univariate filter method used to filter features based on their p-value. Briefly, for each feature, the distribution of percent inhibition values was assessed. If normal, a t-test was performed to yield a p-value. Otherwise, a Kolmogorov-Smirnov test was run. Features were ranked by p-value, and the top 100 features or less that met the 0.05 p-value cutoff were retained for further analysis. The 100 or less features from the first method were then input into Random Forests [20], a multivariate feature selection method based on decision trees. In this phase of the analysis, Random Forests was used to select 10 features for input into the binary classifier.

SVMs are widely used in bioinformatics and other applications of supervised learning. SVMs are used to find a hyperplane that maximizes the margin between the two classes of compounds in *n*-dimensional space, where in this analysis, *n* corresponds to the number of features selected using Random Forests. Initial classification was performed using a nonlinear Radial Basis Function (RBF) kernel, with a cost of 1 and a gamma of 0.

After selecting the model methods, the second phase of the analysis involved optimizing the model parameters. The 10 splits of 5-fold cross validation were then used with the model methods to sweep over the number of features from 2 to 50. Optimal performance was achieved with 45 features. To avoid overfitting and make the model generalizable to future compounds, we selected the minimum number of features whose model yielded an accuracy within one standard deviation of the performance obtained when using 45 features. Thus we selected 21 as the number of features to select for the final model.

Using the full dataset, SVM cost and gamma parameters were then tuned. Briefly, gamma is a parameter that affects the size of the hyperplane in an SVM, while cost is a penalizing measure for having a sample on the wrong side of the hyperplane. Tuning yielded an optimal cost of 2 and a gamma of 2^−4^ using an RBF kernel.

### Model performance

**Figure 2:**
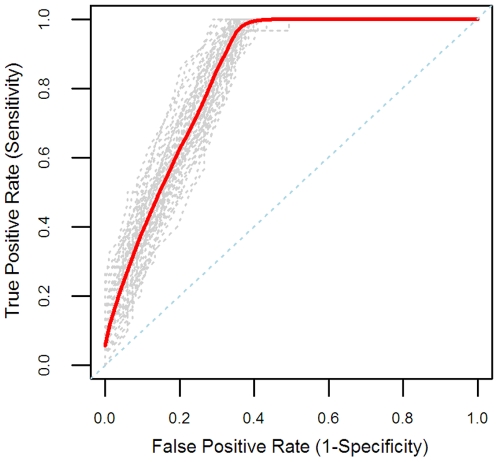
Receiver operating characteristic (ROC) curves for cross-validated assessment of final model performance. Shown are ROC curves for each of the 50 splits of 10-fold cross validation (grey) and overall average ROC curve (red), based on SVM predictions. AUC performance ranged from 0.81 to 0.89 across the 50 splits with an average AUC of 0.84+/−0.021.

Final model performance was based on a re-split of the data into 50 random splits of 10-fold cross validation. Using the final model methods and optimized parameters, the 500 iterations yielded a cross-validated estimate with an overall classification accuracy of 85% (standard deviation 1.8%), sensitivity of 68% (standard deviation 5.0%), and a specificity of 91% (standard deviation 2.0%). A receiver operating characteristic (ROC) curve of the cross-validated and overall mean performance is shown in Figure 2.

### Model kinases

**Figure 3:**
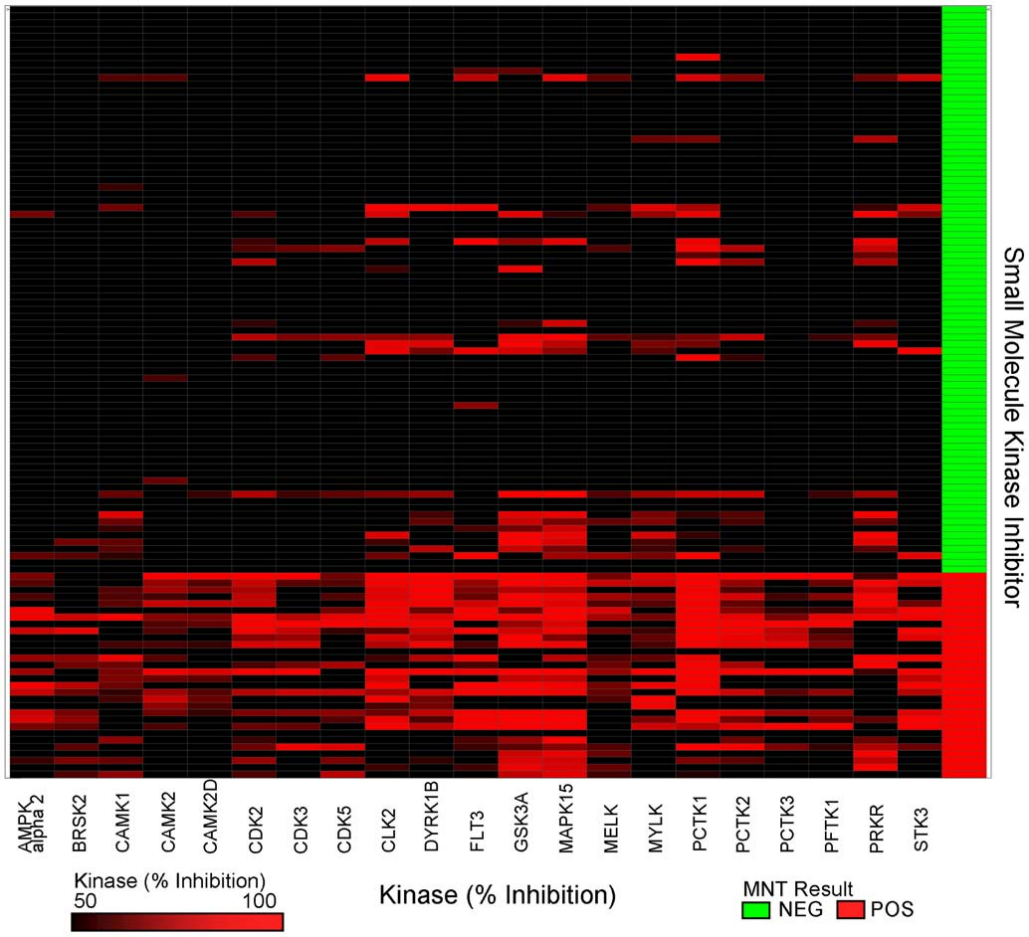
Heat map of kinase inhibition of the 21 kinases selected by mathematical modeling. The 113 SMKIs used in the analysis are classified as micronucleus positive (red, on the bottom) or negative (green) and are located along the y-axis on the right side of the figure. The 21 kinases are located along the x-axis. If inhibited below 50%, the corresponding area is marked black, whereas kinase inhibition between 50–100% is represented by a gradient of dark to bright red.

From the 50 splits of 10-fold cross validation used to assess final model performance, the frequency that each feature was selected as significant in Random Forests was tabulated. The features were then ranked, and the top 21 most frequently-selected kinases were chosen as the model kinase profile. The 21 model kinases are CAMK1 (NP_003647.1), CAMK2A (NP_741960.1), CAMK2D (AAD20442.1), DYRK1B (NP_004705.1), MAPK15 (NP_620590.2), PCTK1 (NP_006192.1), PCTK2 (CAA47004.1), PCTK3 (NP_002587.2), PFTK1 (NP_036527.1), CDK2 (NP_001789.2), CDK3 (NP_001249.1), CDK5 (NP_004926.1), GSK3A (NP_063937.2), CLK2 (NP_003984.2), MELK (NP_055606.1), BRSK2 (NP_003948.2), STK3 (NP_006272.2), MYLK (NP_444254.3), FLT3 (NP_004110.2), EIF2AK2 (NP_002750.1), and PRKAA2 (NP_006243.2). Table 1 lists the 21 kinases and the frequency that each was selected as significant in this phase of the analysis. A heatmap of the percent inhibition values against these 21 kinases is shown in Figure 3 and demonstrates a clear enrichment of kinase inhibition for SMKIs with MNT positive results.

**Table 1:** 21 kinases selected by mathematical modeling.

Entrez Gene Symbol	Entrez Gene Accession	Incidence of Selection	% Selected	q-value
CAMK2A	NP_741960.1	500	100	1.82^−04^
CAMK2D	AAD20442.1	500	100	6.44^−03^
DYRK1B	NP_004705.1	500	100	4.31^−05^
MAPK15	NP_620590.2	500	100	4.81^−07^
PCTK2	CAA47004.1	499	99.8	2.00^−05^
PFTK1	NP_036527.1	498	99.6	1.27^−03^
PCTK1	NP_006192.1	492	98.4	2.00^−05^
PCTK3	NP_002587.2	492	98.4	4.46^−03^
CDK2	NP_001789.2	482	96.4	9.85^−05^
GSK3A	NP_063937.2	447	89.4	6.80^−06^
CDK3	NP_001249.1	438	87.6	8.78^−03^
CLK2	NP_003984.2	431	86.2	2.24^−05^
MELK	NP_055606.1	377	75.4	1.02^−03^
BRSK2	NP_003948.2	367	73.4	1.93^−02^
CAMK1	NP_003647.1	300	60.0	1.37^−02^
STK3	NP_006272.2	282	56.4	1.31^−03^
MYLK	NP_444254.3	263	52.6	4.20^−02^
CDK5	NP_004926.1	254	50.8	6.59^−03^
FLT3	NP_004110.2	241	48.2	4.31^−05^
EIF2AK2	NP_002750.1	217	43.4	4.84^−02^
PRKAA2	NP_006243.2	214	42.8	3.32^−03^

21 model kinases identified by frequency of selection in final phase of model assessment. Incidence of selection and percent selected values were based on 50 splits of 10-fold cross validation in the final phase of assessing model performance. Q-values were calculated from p-values from the Kolmogorov-Smirnov/T-test algorithm in the first phase of feature selection, as described in Results – Model methods.

### Assessment of kinases

To verify the statistical significance of the kinases, a dropout experiment was run using the preprocessed dataset, minus the 21 model kinases. Using the same methods and 50 splits of 10-fold cross validation, the performance of the modified dataset (113 compounds×264 kinases) was assessed. The dropout model yielded an accuracy of 78% (standard deviation 2.4%), sensitivity of 54% (standard deviation 5.9%), and specificity of 87% (standard deviation 2.0%). All performance metrics for the dropout model yielded values at least one standard deviation worse than the original model, demonstrating the significance of the 21 identified kinases. Additionally, to address multiple comparisons concerns, the q-values were calculated for all model kinases. The FDR, or False Discovery Rate [21], estimates the expected proportion of false positives in the data, and in this case, was based on the p-values derived from the Kolmogorov-Smirnov/T-test algorithm on all 290 kinases across all 113 compounds. Q-values, which represent the minimum FDR at which each feature may be called significant, were then calculated using the “qvalue” package in R [22]. At an FDR of 0.05, 73 kinases of 290 may be called significant, including all model kinases. At an FDR of 0.01, 21 kinases may be called significant, although this includes only 10 of the model kinases. Q-values for all model kinases are listed in Table 1. This result is expected since the kinases were selected based on both the filtering of feature selection one and the multivariate criterion of FS2.

To verify the biological significance of the kinases, a review of literature was performed to find studies that might prove or suggest a mechanistic link between the model kinases and mitosis or genetic toxicological damage. The majority of the kinases selected by this analysis (12/21) are members of the CMGC kinase family which is known to be involved with the control of cell proliferation. The cyclin dependent kinases (CDKs) are a family of CMGC kinases that have been associated with mitosis and the cell cycle, and generally speaking, bind with cyclins during the various phases of mitosis. While the three CDKs (2,3, & 5) in the model all appear to have inhibition that is specific to the MNT positive compounds (Figure 3) and are members of a family of kinases known to have roles in mitosis, only CDK2 has supporting literature making it biologically relevant to chromosomal damage.

Little is known about the cellular function of the other CMGC members of the CDK kinase family, such as PFTAIRE (PFTK1) and PCTAIREs 1–3 (PCTKs). From this family, PFTK1 is the only kinase with literature supporting its biological relevance. PFTK1 is a CDK2-related protein kinase which has been reported to phosphorylate the tumor suppressor Rb and interact with p21, suggesting that PFTK1 is involved in cell cycle regulation [23]. The activity of PCTK1 is cell-cycle dependent and displays a peak in the S and G2 phases [24].

Selected kinases in the second largest group (7/21) are members of the calmodulin mediated kinase (CAMK) family, of which only MYLK has been reported to interact with chromosomes. The smooth muscle myosin light chain kinase (smMLCK or MYLK), which facilitates the movement of anaphase chromosomes through its involvement with actin and myosin [25], is the sole kinase from this family with reported association with chromosome kinetics or the cell cycle, which has also been reported to induce spindle disruptions leading to metaphase arrest and chromosome defects [26].

## Discussion

We applied a statistical modeling framework to identify a panel of kinases that are predictive of a positive micronucleus test result, a sign of potential chromosomal damage. To our knowledge, this approach is the first application of a computational method to correlate high-throughput kinase screening results with a toxicological endpoint. The described mathematical model is capable of predicting MNT results correctly 85% of the time based solely on compound inhibition profiles against 21 kinases. The model presented herein indicates that chromosomal damage induced by many of the tested small molecule kinase inhibitors (SMKIs) correlates to their kinase inhibition profiles and that this knowledge can be used to design compounds with improved safety profiles at earlier stages of drug discovery. While the 21 kinases identified in this analysis are statistically significant for our given dataset, our understanding of their mechanistic roles in chromosomal segregation and mitosis is still in its early stages. A heatmap of the inhibition values against these 21 kinases (Figure 3) displays a general pattern: SMKIs that are MNT negative tend to inhibit the 21 kinases much less frequently than the SMKIs that are MNT positive.

While some kinases selected by the model have a known function in cell cycle or chromosomal segregation, others have an unrelated or unknown role. The majority of the kinases chosen are members of the CMGC kinase family, which is known to be involved with the control of cell proliferation. While the role of CDK2 is well-documented, the biological relevance of CDK3 and CDK5 is less clear. CDK3 was identified to complex with cyclin C and phosphorlyate the retinoblastoma tumor suppressor protein [27] and mediates the G1-S transition of the cell cycle [28]. However, there are no reports of its involvement with chromosomal segregation. CDK5 has been described as an unusual member of the CDK family because it has little known role in cellular proliferation and is activated by non-cyclin proteins [29]. The three CDKs (2,3, & 5) chosen all appear to have inhibition that is specific to the MNT positive compounds (Figure 3), and are members of a family of kinases known to have roles in mitosis, thus it is plausible that their inhibition could cause aberrant mitosis and errors in chromosomal segregation. Other model kinases selected from the CMGC family are not known to have a clear role in chromosomal segregation. Collectively, it appears that inhibition of the PCTK kinases (PCTAIREs 1–3) strongly associates with chromosomal damage though this information has not been previously published. The family of MAPKs is well-known to have a fundamental role in mitosis and cell cycle control [30], and are involved with chromosome damage and micronucleus formation [31], though this is the first report placing MAPK15 in such a role. Glycogen synthase kinase 3 alpha (GSK3a) has been reported to be involved with chromosome alignment and cytokinesis [32]–[35], thus its selection by mathematical modeling is not unanticipated. However, GSK3a is one of the more promiscuous kinases selected in the mathematical model, with a large number of micronucleus negative compounds also inhibiting the kinase above 80% at the 10 µM concentration (Figure 3). These data contrast with reported literature, as they suggest that combinations of kinase inhibition, rather than just GSKa alone, may be required for chromosome damage. Model kinases in the calmodulin mediated kinase (CAMK) family were the most specific with regard to their inhibition of micronucleus positive compounds (Figure 3) and were repeatedly chosen by the model, yet the majority, including CAMK1a, CAMK2a and 2d, MELK, BRSK2, and PRKAA2, do not have any reported function involving mitotic chromosome dynamics. The two remaining kinases are FLT3 and MST1, neither of which have any known role in either chromosomal segregation or mitosis. FLT3 has been reported to associate with acute lyphoblastic leukemia [36] and chromosomal instability in the form of hyperdiploid aneuploidy observed in the same disease [37], but its inhibition has not been linked to the missegregation of chromosomes. MST1, also known as STK3, has been identified to be a substrate of caspases and play a role in apoptosis [38].

Besides lack of validation of the mechanistic relevance of some model kinases, additional experimental limitations are that kinase-independent mechanisms of micronucleus formation exist while others are based upon analysis parameters. In any analysis, the robustness of modeling complex endpoints is limited by the training dataset. Care was taken in selecting which compounds to include and were chosen by judiciously sampling our internal kinase inhibitor library for SMKIs representative of broad scaffolds and designed for a variety of kinase targets. Nevertheless, data were limited to past and current kinase projects at Roche and may not reflect future efforts. Another potential concern with this approach is that not all kinases are available in the Ambit competition binding assay. At the time of the analysis, only 290 of the 518 protein kinases were tested. While the 290 kinases account for a large portion of the kinome and do not appear to miss large branches of the kinome tree (data not shown), current panels are more comprehensive and will likely be more complete in the future. In addition to the inherent limitations of the training set, the modeling approach does not necessarily identify all kinases that are highly predictive of chromosome damage. As an example, several kinases were statistically significant in FS1 but were not selected by the FS methods. This is a challenge often observed while analyzing large data sets [39]–[41]: features that correlate individually with the endpoint are not chosen because they may be correlated with other features that are more highly correlated with the endpoint, and thus these features become redundant. Said differently, it is often the case that there is more than one set of features that is highly predictive of the endpoint. Such observations have been made frequently in other areas of biological research where the number of features outnumbers the sample number, e.g. microarray studies [39]–[41].

Despite limitations, the strengths of this method lie in its utility. At Roche and other pharmaceutical companies, SMKIs are designed to inhibit a kinase that has a known role in the pathway or disease of concern. While hundreds of compounds may be developed that strongly bind to their target kinase, it is not often clear whether inhibiting other, non-targeted kinases will affect the success of the compound in further stages of the pipeline, especially in toxicological studies, where many compounds often fail. In the process of building the model, we found that promiscuous SMKIs often tested positive for micronuclei formation (Figure S1), and this was greatly enriched through model development (Figure 3). While general promiscuity may be a relatively good marker for determining the outcome of a micronucleus assay, there are examples where it isn't (Figure S1), suggesting that specific inhibition of particular kinases is of relevance. Providing information to medicinal chemists early in the lead identification/optimization process, beyond just general guidance of promiscuity, is critical to the success of such a strategy, as it provides direction for development as well as compound prioritization for additional development. Knowledge of these 21 kinases within Roche has been of assistance in designing SMKIs where a decrease in genotoxicity has been observed for this compound class.

While the framework presented here provides a robust method for identifying kinases correlated to genotoxicity, causality must be addressed by other means, along with concerns about indirect mechanisms of action and kinases not included in the dataset. There are several kinases that are not solely inhibited by MNT positive compounds, including CLK2, FLT3, GSK3a, MAPK15, PCTK1, and EIF2AK2. This raises the question of whether their inhibition specifically causes the micronucleus formation or if their inhibition requires the inhibition of other kinases for the induction of chromosome damage. Because there is high sequence homology amongst kinases in the ATP binding pocket, it is possible that some of the selected kinases do not cause chromosomal damage, but instead are correlated with the inhibition of others that do. Alternatively, there a number of MNT positive compounds that do not inhibit any of the kinases chosen in the model. Possible explanations include: first, a number of mechanisms independent of kinase inhibition can influence mitotic chromosome dynamics and second, kinases, which have not been screened in this study, may influence the outcome of the micronucleus result.

Development of SMKIs that carry a genotoxic liability can occur, though the generation of additional data demonstrating that the mechanism of action occurs in a non-DNA reactive, threshold-observable manner, is often necessary to appease regulatory agencies. These additional developmental complexities are best avoided, as they are expensive, time consuming, and no guarantee exists that attrition due to chromosomal damage will be avoided. Thus, many companies prefer to spend time and resources during lead optimization to identify compounds free of such liabilities, rather than risk failure due to later-stage attrition. The use of mathematical modeling to better understand what underlies such toxicities is one of the first steps in designing drugs free of these particular liabilities.

Additional studies can shed light on the underlying pathways possibly connecting the model kinases, as it is clear that all are involved in some larger biological network and should not be considered as independent features. Experimental studies can help to confirm possible connections. As a basis for such future investigations, our methodology provides a starting point for biological hypothesis generation, in addition to its utility as a computational model for predicting genotoxicity.

## Materials and Methods

### Compounds

The 113 compounds used in this project were synthesized internally or purchased from Sigma Chemical company (St. Louis, MO). The structural diversity of the 113 compound training set was assessed by representing each compound with Extended Connectivity Fingerprints (ECFP) in Pipeline Pilot 6.0 [42], a molecular characterization of compounds as a 2-dimensional fingerprint. ECFP for each compound was used as input for principal components analysis (PCA), as shown in Figure 1.

### Kinase inhibition

All 113 compounds were sent to Ambit Biosciences (San Diego, CA) for kinase selectivity analysis against 317 kinases using KINOMEscan assays. These 317 kinases cover a large and diverse portion of the human kinome. For each kinase in this high-throughput competition binding screen, ligand-bound kinase quantities are measured in the presence and absence of the compound. Input values for this project are reported in terms of percent inhibition (%) for each compound against each of the 317 kinases. These measurements provide a means for identifying on-target and off-target kinases, as well as for quantifying the selectivity or promiscuity of an SMKI. This is performed in a cell free binding assay which is used as a surrogate for cellular kinase inhibition, which can be influenced by physical-chemical properties (solubility and permeability) that may impact intracellular concentrations and kinase inhibition.

### *In vitro* micronucleus test

The *in vitro* micronucleus assay was conducted according to a previously published protocol [43]. Briefly, the established permanent mouse lymphoma cell line L5178Y *tk^+/−^* (ATCC CRL 9518) growing in suspension was obtained from Covance Laboratories Ltd. (Harrogate, UK). The top dose for evaluation was generally selected to observe acceptable toxicity (decrease of the relative cell count (RCC) below 50%) or clear signs of precipitation in the aqueous medium. Micronucleus results obtained when the RCC falls below 40% are not interpreted as this exceeds the cytotoxicity cut-off. Soluble and non-toxic compounds are evaluated up to a maximal dose level of 5000 µg/mL or 10 mM whichever is lower. The cell cultures were exposed to the test compound for 24 h and harvested either immediately or following a 24 h recovery period in case of cell cycle arrest. For assessment of cytotoxicity cell numbers are scored at harvest with the use of a Coulter Counter and relative cell counts (RCC, as % negative control) were calculated (population doublings and cell morphology were assessed in parallel). 1000 cells per dose were scored with a magnification of 1000× and micronuclei were evaluated according to previously described criteria [44]. A compound is considered to induce a significant level of micronuclei, and thus yield a positive MNT result, if one or more concentrations show at least a 2% frequency of micronucleated cells in either of the two testing regimens (generally corresponding to a 2.5 fold increase over historical controls). Methylmethanesulfonate (15 µg/ml) is used as a micronucleus positive control.

MNT assay results were ultimately considered binary, with a positive result corresponding to toxicity and a negative result to non-toxicity. While some compounds were easily classified, others required reclassification because of experimental parameters. Because Ambit data was measured at a 10 µM concentration for all SMKIs, positive micronucleus results that occurred above this 10 µM cutoff could be due to kinase inhibition that would not be reflected in the kinase inhibition assay results. Thus, to better correlate the kinase inhibition to micronucleus positive results, compounds identified to be micronucleus positive above this threshold were reclassified as negative.

### Dataset

The study was based on a training set of 113 internal SMKIs. To make the model generalizable to the prediction of future compounds, a large and structurally dissimilar group of SMKIs was selected. The training set compounds were chosen to represent a diversity of molecular structures, physicochemical properties, and kinase targets. Each compound was assessed for chromosomal damage, a sign of potential toxicity, using an *in vitro* micronucleus test. Additionally, each compound was screened in a competition binding assay to quantify inhibition of 317 kinases.

Preprocessing was performed prior to employing machine learning methods. From the 317 kinase panel, mutant kinases were removed from the dataset as their mechanistic function would be difficult to interpret. Additional kinases were removed because their percent inhibition level, usually less than 50% at 10 uM, did not differ among the 113 compounds, making them uninformative when separating positive from negative MNT results.

### Model-building framework

**Figure 4:**
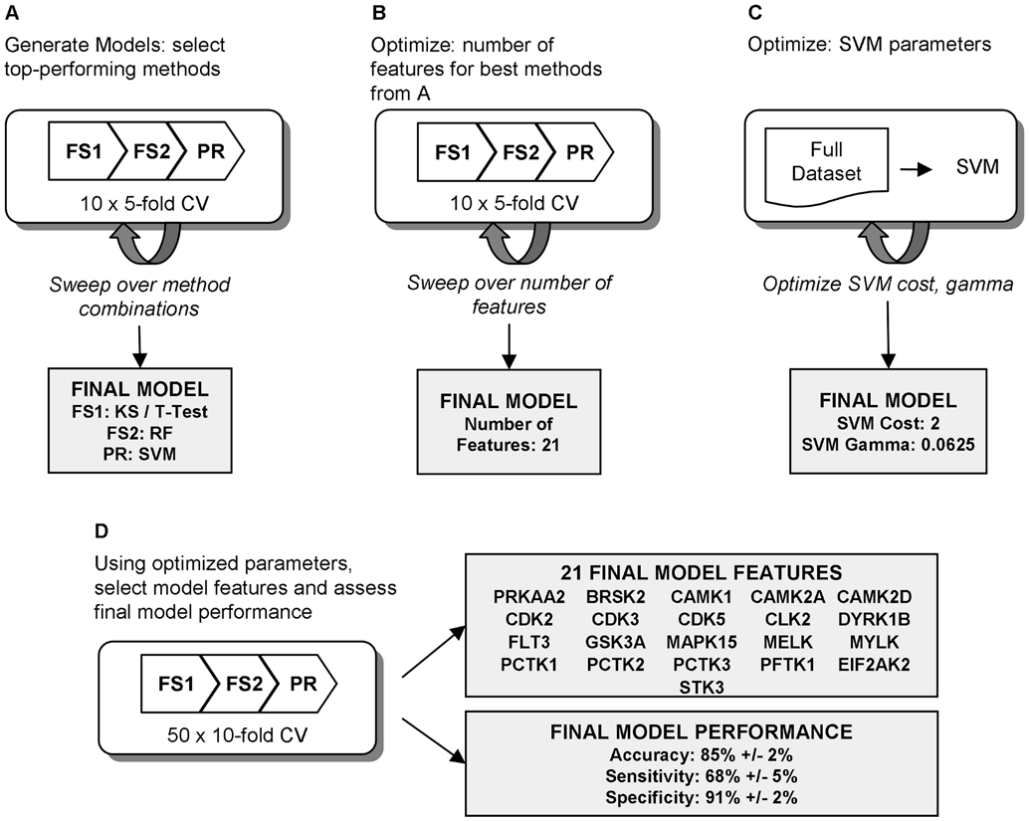
Framework for building, optimizing, and assessing mathematical model. The computational framework for identifying a model first starts by sweeping through combinations of univariate and multivariate feature selection methods, followed by a pattern recognition method. The best performing combination of methods is selected for the final model (A). In the optimization phases, the optimal number of features to use in the final model (B) and tuned parameters of the pattern recognition method (C) are identified. In the final phase of the framework (D), the model methods from A and the optimized parameters from B and C are used to identify the final model features and assess final model performance.

When building a model to predict a binary endpoint, best practices for machine learning recommend using feature selection methods to reduce dimensionality of the data, followed by input into a pattern recognition method. From observation, we have seen that while a selection of certain methods performs well with a given dataset, others methods do not. Similarly, while there are many instances of machine learning methods that perform well, their performance results are not always reproducible with other datasets. In this analysis we present a framework that involves sweeping over variety of methods, which allows the choice of methods to be driven by the data rather than investigator preference, and selecting methods and method parameter based on top-performing results. An overview of the framework is given in Figure 4. Implementation details of the framework, such as choice of programming language and which methods to include, are left to the investigator. This analysis was performed in R version 2.6.2 [45], based on the number of machine learning packages readily available.

### Generation of models

The first phase of the analysis aimed to identify the machine learning methods to be used in the model (Figure 4a). We started by creating 10 random splits of 5-fold stratified cross validation. Briefly, each split randomly grouped the 113 SMKIs into 5 subsets, or folds. Each fold was stratified, meaning that the proportion of MNT positive compounds to MNT negative compounds in each fold roughly reflected that of the full dataset. For each of the *k* folds, *k-1* subsets were used as the training set to build the model, while the remaining subset was used as the test set to estimate performance.

For each fold within each split of data, a combination of two feature selection (FS) methods were run, followed by a binary classifier. FS methods were used to determine which kinases, or features, are likely to correlate most with MNT result. Feature selection methods were separated into two groups: univariate filter methods capable of handling larger input data (FS1), and more computationally-intensive multivariate methods (FS2). FS1 methods consider features independently and are thus less likely to overfit to the given dataset. Such methods include a Kolmogorov-Smirnov/T-test filter, single train error [46], and ReliefF [47]. However, since the FS1 methods do not address redundancy of features, multivariate approaches were employed in FS2 to consider correlation among a given subset of features. FS2 methods included random forests [20], genetic algorithm [48], simulated annealing [48], Gram-Schmidt Orthogonalization [49], and RFE-SVM [50]. The inhibition values against the features chosen in FS2 were then used as input into a pattern recognition (PR) method which predicted a positive or negative result. Classifiers implemented in the PR phase included support vector machines [51], random forests [20], linear discriminant analysis [52], *k*-nearest neighbors [53], partial least squares [54], and principal components analysis [45]. This analysis swept over several combinations of FS1, FS2, and PR methods, although the analysis may include any method that can be implemented within the computational framework.

For each combination of FS1, FS2, and PR, model performance was estimated using 10 splits of 5-fold cross validation. The FS1, FS2, and PR method combination that yielded the greatest accuracy was chosen for model optimization in the next phase of the analysis.

### Model optimization

Once the model methods were selected, the second phase of the analysis aimed to identify the optimal model parameters, namely the number of features to be used in the final model kinase profile, as well as hyperparameters for the binary classifier (Figure 4b and 4c). For the same 10 splits of 5-fold cross validation, FS1, FS2, and PR methods were fixed. First, for each trial of 10×5-fold cross validation, the first part of this phase entailed sweeping over a different number of features to be selected in FS2. This number would ultimately dictate the number of kinases to be used in the final model profile. Similar to selecting model methods in the previous phase, the model number of features was chosen based on which number yielded the lowest error rate. In order to avoid overfitting, the lowest number of features with a mean accuracy within one standard deviation of the optimal number of features' accuracy was chosen for the final model.

The second part of this model optimization involved tuning of the hyperparameters for the optimal PR method. Tuning methodology will be dictated by the optimal PR method selected in the first phase of the model. Our analysis yielded Support Vector Machines (SVM) as the optimal PR method. Thus in this optimization phase of the analysis, we used the full dataset and swept over cost, gamma, and kernel function to identify the optimal SVM hyperparameters to use in the final model. Another example hyperparameter is the number of neighbors *k* used in a vote-based classifier such as *k*-nearest neighbors. Specific parameters depend on which PR method was selected in the previous phase of the analysis.

### Model assessment

The final phase of the analysis aimed to estimate the performance of the final model and identify the model kinase profile (Figure 4d). Ideally, an external set of compounds would be used to validate the model after model optimization. Because such data were not available at the time, model performance was assessed by re-splitting the original dataset into 50 splits of 10-fold cross validation. This number of iterations is much greater than the 10 splits of 5-fold cross validation used when building and tuning the model and provides for a more accurate performance estimate. While more iterations of the model were run, computational costs were manageable as model methods and parameters were already pre-selected. For each fold in each split, the optimal FS1 and FS2 methods selected in the model-generating phase of the analysis were run. The N most informative features were chosen in FS2 for each fold, with N being selected during model optimization. For each fold, the N selected features were then inputted into the optimal PR method with the hyperparameters that were chosen in the model optimization phase.

Final model performance was estimated by calculating mean accuracy, sensitivity, and specificity over all 500 iterations (50 splits of 10-fold). The kinase profile for the final model was then calculated based on frequency of selection during FS2. After tabulating how many times each kinase was selected in the 500 runs, the top N were chosen as the final model set, as they are often selected as informative when separating class labels.

The statistical relevance of kinases in the model profile was verified by performing a dropout experiment. From the 290 kinases, the N final model kinases were removed. The final phase of assessing model performance was then run using 50 splits of 10-fold cross validation with an input data matrix of 113 compounds×(290-N) kinases. Performance results of the dropout experiment were then compared to that of the original model. Additionally, q-values for each of the final model kinases were calculated to assess feature significance based on minimum false discovery rates. The biological relevance of kinases in the model profile was then verified by reviewing literature.

## Supporting Information

Table S1 List of kinases used for analysis.
(0.44 MB DOC)

Figure S1 Kinase inhibition heat map of the 113 small molecule kinase inhibitors assayed for micronuclei and the 290 Ambit panel.
(0.93 MB TIF)
