# Supplementary material for: Identification of a Kinase Profile that Predicts Chromosome Damage Induced by Small Molecule Kinase Inhibitors
Source: PLoS Comput Biol. 2009 Jul 24;5(7):e1000446. doi: 10.1371/journal.pcbi.1000446 (PMC2704959; doi:10.1371/journal.pcbi.1000446)
Supplement: Table S1 — List of kinases used for analysis. (0.44 MB DOC) [file pcbi.1000446.s001.doc]

| **Ambit Gene Symbol** | **Entrez Gene Symbol** | **Accession Number** |
| --- | --- | --- |
| AAK1 | AAK1 | NP_055726.3 |
| ABL1 | ABL1 | [NP_005148.2](http://www.ncbi.nlm.nih.gov/sites/entrez?db=gene&cmd=search&term=25) |
| ABL2 | ABL2 | [NP_005149.3](http://www.ncbi.nlm.nih.gov/sites/entrez?db=gene&cmd=search&term=27) |
| ACVR1 | ACVR1 | [NP_001096.1](http://www.ncbi.nlm.nih.gov/sites/entrez?db=gene&cmd=search&term=90) |
| ACVR1B | ACVR1B | [NP_004293.1](http://www.ncbi.nlm.nih.gov/sites/entrez?db=gene&cmd=search&term=91) |
| ACVR2A | ACVR2A | [NP_001607.1](http://www.ncbi.nlm.nih.gov/sites/entrez?db=gene&cmd=search&term=92) |
| ACVR2B | ACVR2B | [NP_001097.2](http://www.ncbi.nlm.nih.gov/sites/entrez?db=gene&cmd=search&term=93) |
| ACVRL1 | ACVRL1 | [NP_000011.1](http://www.ncbi.nlm.nih.gov/sites/entrez?db=gene&cmd=search&term=94) |
| ADCK3 | CABC1 | [NP_064632.2](http://www.ncbi.nlm.nih.gov/sites/entrez?db=gene&cmd=search&term=56997) |
| ADCK4 | ADCK4 | [NP_079152.3](http://www.ncbi.nlm.nih.gov/sites/entrez?db=gene&cmd=search&term=79934) |
| AKT1 | AKT1 | [NP_005154.2](http://www.ncbi.nlm.nih.gov/sites/entrez?db=gene&cmd=search&term=207) |
| AKT2 | AKT2 | [NP_001617.1](http://www.ncbi.nlm.nih.gov/sites/entrez?db=gene&cmd=search&term=208) |
| AKT3 | AKT3 | [NP_005456.1](http://www.ncbi.nlm.nih.gov/sites/entrez?db=gene&cmd=search&term=10000) |
| ALK | ALK | [NP_004295.2](http://www.ncbi.nlm.nih.gov/sites/entrez?db=gene&cmd=search&term=238) |
| AMPK-alpha1 | PRKAA1 | [BAA36547.1](http://www.ncbi.nlm.nih.gov/sites/entrez?db=gene&cmd=search&term=5562) |
| AMPK-alpha2 | PRKAA2 | [NP_006243.2](http://www.ncbi.nlm.nih.gov/sites/entrez?db=gene&cmd=search&term=5563) |
| ANKK1 | ANKK1 | [NP_848605.1](http://www.ncbi.nlm.nih.gov/sites/entrez?db=gene&cmd=search&term=255239) |
| ARK5 | NUAK1 | [NP_055655.1](http://www.ncbi.nlm.nih.gov/sites/entrez?db=gene&cmd=search&term=9891) |
| ASK1 | MAP3K5 | [NP_005914.1](http://www.ncbi.nlm.nih.gov/sites/entrez?db=gene&cmd=search&term=4217) |
| AURKA | AURKA | [NP_003591.2](http://www.ncbi.nlm.nih.gov/sites/entrez?db=gene&cmd=search&term=6790) |
| AURKB | AURKB | [AAH00442.2](http://www.ncbi.nlm.nih.gov/sites/entrez?db=gene&cmd=search&term=9212) |
| AURKC | AURKC | [AAC77369.1](http://www.ncbi.nlm.nih.gov/sites/entrez?db=gene&cmd=search&term=6795) |
| AXL | AXL | [NP_001690.2](http://www.ncbi.nlm.nih.gov/sites/entrez?db=gene&cmd=search&term=558) |
| BIKE | BMP2K | [NP_060063.2](http://www.ncbi.nlm.nih.gov/sites/entrez?db=gene&cmd=search&term=55589) |
| BLK | BLK | [NP_001706.2](http://www.ncbi.nlm.nih.gov/sites/entrez?db=gene&cmd=search&term=640) |
| BMPR1A | BMPR1A | [NP_004320.2](http://www.ncbi.nlm.nih.gov/sites/entrez?db=gene&cmd=search&term=657) |
| BMPR2 | BMPR2 | [NP_001195.2](http://www.ncbi.nlm.nih.gov/sites/entrez?db=gene&cmd=search&term=659) |
| BMX | BMX | [NP_001712.1](http://www.ncbi.nlm.nih.gov/sites/entrez?db=gene&cmd=search&term=660) |
| BRAF | BRAF | [NP_004324.2](http://www.ncbi.nlm.nih.gov/sites/entrez?db=gene&cmd=search&term=673) |
| BRK | PTK6 | [NP_005966.1](http://www.ncbi.nlm.nih.gov/sites/entrez?db=gene&cmd=search&term=5753) |
| BRSK1 | BRSK1 | [NP_115806.1](http://www.ncbi.nlm.nih.gov/sites/entrez?db=gene&cmd=search&term=84446) |
| BRSK2 | BRSK2 | [NP_003948.2](http://www.ncbi.nlm.nih.gov/sites/entrez?db=gene&cmd=search&term=9024) |
| BTK | BTK | [NP_000052.1](http://www.ncbi.nlm.nih.gov/sites/entrez?db=gene&cmd=search&term=695) |
| CAMK1 | CAMK1 | [NP_003647.1](http://www.ncbi.nlm.nih.gov/sites/entrez?db=gene&cmd=search&term=8536) |
| CAMK1D | CAMK1D | [NP_065130.1](http://www.ncbi.nlm.nih.gov/sites/entrez?db=gene&cmd=search&term=57118) |
| CAMK1G | CAMK1G | [NP_065172.1](http://www.ncbi.nlm.nih.gov/sites/entrez?db=gene&cmd=search&term=57172) |
| CAMK2A | CAMK2A | [NP_741960.1](http://www.ncbi.nlm.nih.gov/sites/entrez?db=gene&cmd=search&term=815) |
| CAMK2B | CAMK2B | [NP_001211.3](http://www.ncbi.nlm.nih.gov/sites/entrez?db=gene&cmd=search&term=816) |
| CAMK2D | CAMK2D | [AAD20442.1](http://www.ncbi.nlm.nih.gov/sites/entrez?db=gene&cmd=search&term=817) |
| CAMK2G | CAMK2G | [NP_751913.1](http://www.ncbi.nlm.nih.gov/sites/entrez?db=gene&cmd=search&term=818) |
| CAMK4 | CAMK4 | [NP_001735.1](http://www.ncbi.nlm.nih.gov/sites/entrez?db=gene&cmd=search&term=814) |
| CAMKK1 | CAMKK1 | [NP_115670.1](http://www.ncbi.nlm.nih.gov/sites/entrez?db=gene&cmd=search&term=84254) |
| CAMKK2 | CAMKK2 | [NP_006540.3](http://www.ncbi.nlm.nih.gov/sites/entrez?db=gene&cmd=search&term=10645) |
| CDC2L1 | CDC2L1 | [NP_277023.1](http://www.ncbi.nlm.nih.gov/sites/entrez?db=gene&cmd=search&term=984) |
| CDC2L2 | CDC2L2 | [NP_076916.1](http://www.ncbi.nlm.nih.gov/sites/entrez?db=gene&cmd=search&term=728642) |
| CDK11 | CDC2L6 | [NP_055891.1](http://www.ncbi.nlm.nih.gov/sites/entrez?db=gene&cmd=search&term=23097) |
| CDK2 | CDK2 | [NP_001789.2](http://www.ncbi.nlm.nih.gov/sites/entrez?db=gene&cmd=search&term=1017) |
| CDK3 | CDK3 | [NP_001249.1](http://www.ncbi.nlm.nih.gov/sites/entrez?db=gene&cmd=search&term=1018) |
| CDK5 | CDK5 | [NP_004926.1](http://www.ncbi.nlm.nih.gov/sites/entrez?db=gene&cmd=search&term=1020) |
| CDK7 | CDK7 | [NP_001790.1](http://www.ncbi.nlm.nih.gov/sites/entrez?db=gene&cmd=search&term=1022) |
| CDK8 | CDK8 | [NP_001251.1](http://www.ncbi.nlm.nih.gov/sites/entrez?db=gene&cmd=search&term=1024) |
| CDK9 | CDK9 | [NP_004293.1](http://www.ncbi.nlm.nih.gov/sites/entrez?db=gene&cmd=search&term=91) |
| CHEK1 | CHEK1 | [NP_001265.1](http://www.ncbi.nlm.nih.gov/sites/entrez?db=gene&cmd=search&term=1111) |
| CIT | CIT | [NP_009105.1](http://www.ncbi.nlm.nih.gov/sites/entrez?db=gene&cmd=search&term=11113) |
| CLK1 | CLK1 | [AAA61480.1](http://www.ncbi.nlm.nih.gov/sites/entrez?db=gene&cmd=search&term=1195) |
| CLK2 | CLK2 | [NP_003984.2](http://www.ncbi.nlm.nih.gov/sites/entrez?db=gene&cmd=search&term=1196) |
| CLK3 | CLK3 | [NP_003983.2](http://www.ncbi.nlm.nih.gov/sites/entrez?db=gene&cmd=search&term=1198) |
| CLK4 | CLK4 | [NP_065717.1](http://www.ncbi.nlm.nih.gov/sites/entrez?db=gene&cmd=search&term=57396) |
| CSF1R | CSF1R | [NP_005202.2](http://www.ncbi.nlm.nih.gov/sites/entrez?db=gene&cmd=search&term=1436) |
| CSK | CSK | [NP_004374.1](http://www.ncbi.nlm.nih.gov/sites/entrez?db=gene&cmd=search&term=1445) |
| CSNK1A1L | CSNK1A1L | [NP_660204.1](http://www.ncbi.nlm.nih.gov/sites/entrez?db=gene&cmd=search&term=122011) |
| CSNK1D | CSNK1D | [NP_620693.1](http://www.ncbi.nlm.nih.gov/sites/entrez?db=gene&cmd=search&term=1453) |
| CSNK1E | CSNK1E | [NP_001885.1](http://www.ncbi.nlm.nih.gov/sites/entrez?db=gene&cmd=search&term=1454) |
| CSNK1G1 | CSNK1G1 | [NP_071331.2](http://www.ncbi.nlm.nih.gov/sites/entrez?db=gene&cmd=search&term=53944) |
| CSNK1G2 | CSNK1G2 | [NP_001310.2](http://www.ncbi.nlm.nih.gov/sites/entrez?db=gene&cmd=search&term=1455) |
| CSNK1G3 | CSNK1G3 | [NP_004375.2](http://www.ncbi.nlm.nih.gov/sites/entrez?db=gene&cmd=search&term=1456) |
| CSNK2A1 | CSNK2A1 | [NP_001886.1](http://www.ncbi.nlm.nih.gov/sites/entrez?db=gene&cmd=search&term=1457) |
| CSNK2A2 | CSNK2A2 | [NP_001887.1](http://www.ncbi.nlm.nih.gov/sites/entrez?db=gene&cmd=search&term=1459) |
| DAPK1 | DAPK1 | [NP_004929.2](http://www.ncbi.nlm.nih.gov/sites/entrez?db=gene&cmd=search&term=1612) |
| DAPK2 | DAPK2 | [NP_055141.2](http://www.ncbi.nlm.nih.gov/sites/entrez?db=gene&cmd=search&term=23604) |
| DAPK3 | DAPK3 | [NP_001339.1](http://www.ncbi.nlm.nih.gov/sites/entrez?db=gene&cmd=search&term=1613) |
| DCAMKL1 | DCLK1 | [NP_004725.1](http://www.ncbi.nlm.nih.gov/sites/entrez?db=gene&cmd=search&term=9201) |
| DCAMKL2 | DCLK2 | [NP_001035351.3](http://www.ncbi.nlm.nih.gov/sites/entrez?db=gene&cmd=search&term=166614) |
| DCAMKL3 | DCLK3 | [NP_208382.1](http://www.ncbi.nlm.nih.gov/sites/entrez?db=gene&cmd=search&term=85443) |
| DDR1 | DDR1 | [NP_001945.3](http://www.ncbi.nlm.nih.gov/sites/entrez?db=gene&cmd=search&term=780) |
| DDR2 | DDR2 | [CAA52777.1](http://www.ncbi.nlm.nih.gov/sites/entrez?db=gene&cmd=search&term=4921) |
| DLK | MAP3K12 | [NP_006292.2](http://www.ncbi.nlm.nih.gov/sites/entrez?db=gene&cmd=search&term=7786) |
| DMPK | DMPK | [NP_004400.4](http://www.ncbi.nlm.nih.gov/sites/entrez?db=gene&cmd=search&term=1760) |
| DMPK2 | CDC42BPG | [NP_059995.2](http://www.ncbi.nlm.nih.gov/sites/entrez?db=gene&cmd=search&term=55561) |
| DRAK1 | STK17A | [NP_004751.1](http://www.ncbi.nlm.nih.gov/sites/entrez?db=gene&cmd=search&term=9263) |
| DRAK2 | STK17B | [NP_004217.1](http://www.ncbi.nlm.nih.gov/sites/entrez?db=gene&cmd=search&term=9262) |
| DYRK1B | DYRK1B | [NP_004705.1](http://www.ncbi.nlm.nih.gov/sites/entrez?db=gene&cmd=search&term=9149) |
| EGFR | EGFR | [NP_005219.2](http://www.ncbi.nlm.nih.gov/sites/entrez?db=gene&cmd=search&term=1956) |
| EPHA1 | EPHA1 | [NP_005223.3](http://www.ncbi.nlm.nih.gov/sites/entrez?db=gene&cmd=search&term=) |
| EPHA2 | EPHA2 | [NP_004422.2](http://www.ncbi.nlm.nih.gov/sites/entrez?db=gene&cmd=search&term=1969) |
| EPHA3 | EPHA3 | [NP_005224.2](http://www.ncbi.nlm.nih.gov/sites/entrez?db=gene&cmd=search&term=2042) |
| EPHA4 | EPHA4 | [NP_004429.1](http://www.ncbi.nlm.nih.gov/sites/entrez?db=gene&cmd=search&term=2043) |
| EPHA5 | EPHA5 | [NP_004430.3](http://www.ncbi.nlm.nih.gov/sites/entrez?db=gene&cmd=search&term=) |
| EPHA6 | EPHA6 | [NP_001073917.2](http://www.ncbi.nlm.nih.gov/sites/entrez?db=gene&cmd=search&term=285220) |
| EPHA7 | EPHA7 | [NP_004431.1](http://www.ncbi.nlm.nih.gov/sites/entrez?db=gene&cmd=search&term=2045) |
| EPHA8 | EPHA8 | [NP_065387.1](http://www.ncbi.nlm.nih.gov/sites/entrez?db=gene&cmd=search&term=2046) |
| EPHB1 | EPHB1 | [NP_004432.1](http://www.ncbi.nlm.nih.gov/sites/entrez?db=gene&cmd=search&term=2047) |
| EPHB2 | EPHB2 | [NP_059145.2](http://www.ncbi.nlm.nih.gov/sites/entrez?db=gene&cmd=search&term=2048) |
| EPHB3 | EPHB3 | [NP_004434.2](http://www.ncbi.nlm.nih.gov/sites/entrez?db=gene&cmd=search&term=2049) |
| EPHB4 | EPHB4 | [NP_004435.3](http://www.ncbi.nlm.nih.gov/sites/entrez?db=gene&cmd=search&term=2050) |
| ERBB2 | ERBB2 | [NP_001005862.1](http://www.ncbi.nlm.nih.gov/sites/entrez?db=gene&cmd=search&term=2064) |
| ERBB4 | ERBB4 | [NP_001036064.1](http://www.ncbi.nlm.nih.gov/sites/entrez?db=gene&cmd=search&term=2066) |
| ERK1 | MAPK3 | [NP_002737.2](http://www.ncbi.nlm.nih.gov/sites/entrez?db=gene&cmd=search&term=5595) |
| ERK2 | MAPK1 | [NP_620407.1](http://www.ncbi.nlm.nih.gov/sites/entrez?db=gene&cmd=search&term=5594) |
| ERK3 | MAPK6 | [NP_002739.1](http://www.ncbi.nlm.nih.gov/sites/entrez?db=gene&cmd=search&term=5597) |
| ERK4 | MAPK4 | [NP_002738.2](http://www.ncbi.nlm.nih.gov/sites/entrez?db=gene&cmd=search&term=5596) |
| ERK5 | MAPK7 | [NP_002740.2](http://www.ncbi.nlm.nih.gov/sites/entrez?db=gene&cmd=search&term=5598) |
| ERK8 | MAPK15 | [NP_620590.2](http://www.ncbi.nlm.nih.gov/sites/entrez?db=gene&cmd=search&term=225689) |
| FAK | PTK2 | [NP_722560.1](http://www.ncbi.nlm.nih.gov/sites/entrez?db=gene&cmd=search&term=5747) |
| FER | FER | [NP_005237.2](http://www.ncbi.nlm.nih.gov/sites/entrez?db=gene&cmd=search&term=2241) |
| FES | FES | [NP_001996.1](http://www.ncbi.nlm.nih.gov/sites/entrez?db=gene&cmd=search&term=2242) |
| FGFR1 | FGFR1 | [NP_075593.1](http://www.ncbi.nlm.nih.gov/sites/entrez?db=gene&cmd=search&term=2260) |
| FGFR2 | FGFR2 | [NP_075259.2](http://www.ncbi.nlm.nih.gov/sites/entrez?db=gene&cmd=search&term=) |
| FGFR3 | FGFR3 | [NP_000133.1](http://www.ncbi.nlm.nih.gov/sites/entrez?db=gene&cmd=search&term=2261) |
| FGFR4 | FGFR4 | [NP_075252.2](http://www.ncbi.nlm.nih.gov/sites/entrez?db=gene&cmd=search&term=2264) |
| FGR | FGR | [NP_005239.1](http://www.ncbi.nlm.nih.gov/sites/entrez?db=gene&cmd=search&term=2268) |
| FLT1 | FLT1 | [NP_002010.2](http://www.ncbi.nlm.nih.gov/sites/entrez?db=gene&cmd=search&term=2321) |
| FLT3 | FLT3 | [NP_004110.2](http://www.ncbi.nlm.nih.gov/sites/entrez?db=gene&cmd=search&term=2322) |
| FLT3(ITD) | FLT3 | [NP_004110.2](http://www.ncbi.nlm.nih.gov/sites/entrez?db=gene&cmd=search&term=2322) |
| FLT4 | FLT4 | [NP_002011.1](http://www.ncbi.nlm.nih.gov/sites/entrez?db=gene&cmd=search&term=2324) |
| FRK | FRK | [NP_002022.1](http://www.ncbi.nlm.nih.gov/sites/entrez?db=gene&cmd=search&term=2444) |
| FYN | FYN | [NP_694592.1](http://www.ncbi.nlm.nih.gov/sites/entrez?db=gene&cmd=search&term=2534) |
| GAK | GAK | [NP_005246.1](http://www.ncbi.nlm.nih.gov/sites/entrez?db=gene&cmd=search&term=2580) |
| GSK3A | GSK3A | [NP_063937.2](http://www.ncbi.nlm.nih.gov/sites/entrez?db=gene&cmd=search&term=2931) |
| GSK3B | GSK3B | [NP_002084.2](http://www.ncbi.nlm.nih.gov/sites/entrez?db=gene&cmd=search&term=2932) |
| HCK | HCK | [NP_002101.2](http://www.ncbi.nlm.nih.gov/sites/entrez?db=gene&cmd=search&term=3055) |
| HPK1 | MAP4K1 | [NP_001036065.1](http://www.ncbi.nlm.nih.gov/sites/entrez?db=gene&cmd=search&term=11184) |
| IGF1R | IGF1R | [NP_000866.1](http://www.ncbi.nlm.nih.gov/sites/entrez?db=gene&cmd=search&term=3480) |
| IKK-epsilon | IKBKE | [NP_054721.1](http://www.ncbi.nlm.nih.gov/sites/entrez?db=gene&cmd=search&term=9641) |
| INSR | INSR | [NP_000199.2](http://www.ncbi.nlm.nih.gov/sites/entrez?db=gene&cmd=search&term=3643) |
| INSRR | INSRR | [NP_055030.1](http://www.ncbi.nlm.nih.gov/sites/entrez?db=gene&cmd=search&term=3645) |
| IRAK3 | IRAK3 | [NP_009130.1](http://www.ncbi.nlm.nih.gov/sites/entrez?db=gene&cmd=search&term=11213) |
| ITK | ITK | [NP_005537.3](http://www.ncbi.nlm.nih.gov/sites/entrez?db=gene&cmd=search&term=3702) |
| JAK1(JH1domain-catalytic) | JAK1 | [NP_002218.2](http://www.ncbi.nlm.nih.gov/sites/entrez?db=gene&cmd=search&term=3716) |
| JAK2(JH1domain-catalytic) | JAK2 | [NP_004963.1](http://www.ncbi.nlm.nih.gov/sites/entrez?db=gene&cmd=search&term=3717) |
| JAK3(JH1domain-catalytic) | JAK3 | [NP_000206.2](http://www.ncbi.nlm.nih.gov/sites/entrez?db=gene&cmd=search&term=3718) |
| JNK1 | MAPK8 | [NP_002741.1](http://www.ncbi.nlm.nih.gov/sites/entrez?db=gene&cmd=search&term=5599) |
| JNK2 | MAPK9 | [NP_620707.1](http://www.ncbi.nlm.nih.gov/sites/entrez?db=gene&cmd=search&term=5601) |
| JNK3 | MAPK10 | [NP_002744.1](http://www.ncbi.nlm.nih.gov/sites/entrez?db=gene&cmd=search&term=5602) |
| KIT | KIT | [NP_000213.1](http://www.ncbi.nlm.nih.gov/sites/entrez?db=gene&cmd=search&term=3815) |
| LATS1 | LATS1 | [NP_004681.1](http://www.ncbi.nlm.nih.gov/sites/entrez?db=gene&cmd=search&term=9113) |
| LATS2 | LATS2 | [NP_055387.1](http://www.ncbi.nlm.nih.gov/sites/entrez?db=gene&cmd=search&term=26524) |
| LCK | LCK | [NP_005347.3](http://www.ncbi.nlm.nih.gov/sites/entrez?db=gene&cmd=search&term=3932) |
| LIMK1 | LIMK1 | [NP_002305.1](http://www.ncbi.nlm.nih.gov/sites/entrez?db=gene&cmd=search&term=3984) |
| LIMK2 | LIMK2 | [NP_005560.1](http://www.ncbi.nlm.nih.gov/sites/entrez?db=gene&cmd=search&term=3985) |
| LKB1 | STK11 | [NP_000446.1](http://www.ncbi.nlm.nih.gov/sites/entrez?db=gene&cmd=search&term=6794) |
| LOK | STK10 | [NP_005981.3](http://www.ncbi.nlm.nih.gov/sites/entrez?db=gene&cmd=search&term=6793) |
| LTK | LTK | [NP_996844.1](http://www.ncbi.nlm.nih.gov/sites/entrez?db=gene&cmd=search&term=4058) |
| LYN | LYN | [NP_002341.1](http://www.ncbi.nlm.nih.gov/sites/entrez?db=gene&cmd=search&term=4067) |
| MAP3K4 | MAP3K4 | [NP_005913.2](http://www.ncbi.nlm.nih.gov/sites/entrez?db=gene&cmd=search&term=4216) |
| MAP4K3 | MAP4K3 | [NP_003609.2](http://www.ncbi.nlm.nih.gov/sites/entrez?db=gene&cmd=search&term=8491) |
| MAP4K4 | MAP4K4 | [NP_663719.1](http://www.ncbi.nlm.nih.gov/sites/entrez?db=gene&cmd=search&term=9448) |
| MAP4K5 | MAP4K5 | [NP_006566.2](http://www.ncbi.nlm.nih.gov/sites/entrez?db=gene&cmd=search&term=11183) |
| MAPKAPK2 | MAPKAPK2 | [NP_116584.2](http://www.ncbi.nlm.nih.gov/sites/entrez?db=gene&cmd=search&term=9261) |
| MAPKAPK5 | MAPKAPK5 | [NP_003659.2](http://www.ncbi.nlm.nih.gov/sites/entrez?db=gene&cmd=search&term=8550) |
| MARK1 | MARK1 | [NP_061120.3](http://www.ncbi.nlm.nih.gov/sites/entrez?db=gene&cmd=search&term=4139) |
| MARK2 | MARK2 | [NP_059672.2](http://www.ncbi.nlm.nih.gov/sites/entrez?db=gene&cmd=search&term=2011) |
| MARK3 | MARK3 | [NP_002367.4](http://www.ncbi.nlm.nih.gov/sites/entrez?db=gene&cmd=search&term=4140) |
| MARK4 | MARK4 | [NP_113605.2](http://www.ncbi.nlm.nih.gov/sites/entrez?db=gene&cmd=search&term=57787) |
| MEK1 | MAP2K1 | [NP_002746.1](http://www.ncbi.nlm.nih.gov/sites/entrez?db=gene&cmd=search&term=5604) |
| MEK2 | MAP2K2 | [NP_109587.1](http://www.ncbi.nlm.nih.gov/sites/entrez?db=gene&cmd=search&term=5605) |
| MEK3 | MAP2K3 | [NP_002747.2](http://www.ncbi.nlm.nih.gov/sites/entrez?db=gene&cmd=search&term=5606) |
| MEK4 | MAP2K4 | [NP_003001.1](http://www.ncbi.nlm.nih.gov/sites/entrez?db=gene&cmd=search&term=6416) |
| MEK6 | MAP2K6 | [NP_002749.2](http://www.ncbi.nlm.nih.gov/sites/entrez?db=gene&cmd=search&term=5608) |
| MELK | MELK | [NP_055606.1](http://www.ncbi.nlm.nih.gov/sites/entrez?db=gene&cmd=search&term=9833) |
| MERTK | MERTK | [AAB60430.1](http://www.ncbi.nlm.nih.gov/sites/entrez?db=gene&cmd=search&term=10461) |
| MET | MET | [NP_000236.2](http://www.ncbi.nlm.nih.gov/sites/entrez?db=gene&cmd=search&term=4233) |
| MKNK1 | MKNK1 | [CAI14764.1](http://www.ncbi.nlm.nih.gov/sites/entrez?db=gene&cmd=search&term=8569) |
| MKNK2 | MKNK2 | [AAF17226.1](http://www.ncbi.nlm.nih.gov/sites/entrez?db=gene&cmd=search&term=2872) |
| MLCK | MLCK | [NP_872299.2](http://www.ncbi.nlm.nih.gov/sites/entrez?db=gene&cmd=search&term=91807) |
| MLK1 | MAP3K9 | [NP_149132.2](http://www.ncbi.nlm.nih.gov/sites/entrez?db=gene&cmd=search&term=4293) |
| MLK2 | MAP3K10 | [NP_002437.2](http://www.ncbi.nlm.nih.gov/sites/entrez?db=gene&cmd=search&term=4294) |
| MLK3 | MAP3K11 | [NP_002410.1](http://www.ncbi.nlm.nih.gov/sites/entrez?db=gene&cmd=search&term=4296) |
| MRCKA | CDC42BPA | [NP_003598.2](http://www.ncbi.nlm.nih.gov/sites/entrez?db=gene&cmd=search&term=8476) |
| MRCKB | CDC42BPB | [NP_006026.3](http://www.ncbi.nlm.nih.gov/sites/entrez?db=gene&cmd=search&term=9578) |
| MST1 | STK4 | [NP_006273.1](http://www.ncbi.nlm.nih.gov/sites/entrez?db=gene&cmd=search&term=6789) |
| MST2 | STK3 | [NP_006272.1](http://www.ncbi.nlm.nih.gov/sites/entrez?db=gene&cmd=search&term=6788) |
| MST3 | STK24 | [NP_003567.2](http://www.ncbi.nlm.nih.gov/sites/entrez?db=gene&cmd=search&term=8428) |
| MST4 | MST4 | [NP_057626.2](http://www.ncbi.nlm.nih.gov/sites/entrez?db=gene&cmd=search&term=51765) |
| MUSK | MUSK | [NP_005583.1](http://www.ncbi.nlm.nih.gov/sites/entrez?db=gene&cmd=search&term=4593) |
| MYLK | MYLK | [NP_444254.3](http://www.ncbi.nlm.nih.gov/sites/entrez?db=gene&cmd=search&term=4638) |
| MYLK2 | MYLK2 | [NP_149109.1](http://www.ncbi.nlm.nih.gov/sites/entrez?db=gene&cmd=search&term=85366) |
| MYO3A | MYO3A | [NP_059129.3](http://www.ncbi.nlm.nih.gov/sites/entrez?db=gene&cmd=search&term=53904) |
| MYO3B | MYO3B | [NP_001077084.1](http://www.ncbi.nlm.nih.gov/sites/entrez?db=gene&cmd=search&term=140469) |
| NDR2 | STK38L | [NP_055815.1](http://www.ncbi.nlm.nih.gov/sites/entrez?db=gene&cmd=search&term=23012) |
| NEK1 | NEK1 | [NP_036356.1](http://www.ncbi.nlm.nih.gov/sites/entrez?db=gene&cmd=search&term=4750) |
| NEK2 | NEK2 | [NP_002488.1](http://www.ncbi.nlm.nih.gov/sites/entrez?db=gene&cmd=search&term=4751) |
| NEK5 | NEK5 | [NP_954983.1](http://www.ncbi.nlm.nih.gov/sites/entrez?db=gene&cmd=search&term=341676) |
| NEK6 | NEK6 | [NP_055212.2](http://www.ncbi.nlm.nih.gov/sites/entrez?db=gene&cmd=search&term=10783) |
| NEK7 | NEK7 | [NP_598001.1](http://www.ncbi.nlm.nih.gov/sites/entrez?db=gene&cmd=search&term=140609) |
| NEK9 | NEK9 | [NP_149107.3](http://www.ncbi.nlm.nih.gov/sites/entrez?db=gene&cmd=search&term=91754) |
| NLK | NLK | [NP_057315.3](http://www.ncbi.nlm.nih.gov/sites/entrez?db=gene&cmd=search&term=51701) |
| p38-alpha | MAPK14 | [NP_620581.1](http://www.ncbi.nlm.nih.gov/sites/entrez?db=gene&cmd=search&term=1432) |
| p38-beta | MAPK11 | [NP_002742.3](http://www.ncbi.nlm.nih.gov/sites/entrez?db=gene&cmd=search&term=5600) |
| p38-gamma | MAPK12 | [AAB40118.1](http://www.ncbi.nlm.nih.gov/sites/entrez?db=gene&cmd=search&term=6300) |
| PAK1 | PAK1 | [NP_002567.3](http://www.ncbi.nlm.nih.gov/sites/entrez?db=gene&cmd=search&term=5058) |
| PAK2 | PAK2 | [NP_002568.2](http://www.ncbi.nlm.nih.gov/sites/entrez?db=gene&cmd=search&term=5062) |
| PAK3 | PAK3 | [NP_002569.1](http://www.ncbi.nlm.nih.gov/sites/entrez?db=gene&cmd=search&term=5063) |
| PAK4 | PAK4 | [NP_001014834.1](http://www.ncbi.nlm.nih.gov/sites/entrez?db=gene&cmd=search&term=10298) |
| PAK6 | PAK6 | [NP_064553.1](http://www.ncbi.nlm.nih.gov/sites/entrez?db=gene&cmd=search&term=56924) |
| PAK7 | PAK7 | [NP_065074.1](http://www.ncbi.nlm.nih.gov/sites/entrez?db=gene&cmd=search&term=57144) |
| PCTK1 | PCTK1 | [NP_006192.1](http://www.ncbi.nlm.nih.gov/sites/entrez?db=gene&cmd=search&term=5127) |
| PCTK2 | PCTK2 | [CAA47004.1](http://www.ncbi.nlm.nih.gov/sites/entrez?db=gene&cmd=search&term=5128) |
| PCTK3 | PCTK3 | [NP_002587.2](http://www.ncbi.nlm.nih.gov/sites/entrez?db=gene&cmd=search&term=5129) |
| PDGFRA | PDGFRA | [NP_006197.1](http://www.ncbi.nlm.nih.gov/sites/entrez?db=gene&cmd=search&term=5156) |
| PDGFRB | PDGFRB | [NP_002600.1](http://www.ncbi.nlm.nih.gov/sites/entrez?db=gene&cmd=search&term=5159) |
| PDPK1 | PDPK1 | [NP_002604.1](http://www.ncbi.nlm.nih.gov/sites/entrez?db=gene&cmd=search&term=5170) |
| PFTK1 | PFTK1 | [NP_036527.1](http://www.ncbi.nlm.nih.gov/sites/entrez?db=gene&cmd=search&term=5218) |
| PHKG1 | PHKG1 | [NP_006204.1](http://www.ncbi.nlm.nih.gov/sites/entrez?db=gene&cmd=search&term=5260) |
| PHKG2 | PHKG2 | [NP_000285.1](http://www.ncbi.nlm.nih.gov/sites/entrez?db=gene&cmd=search&term=5261) |
| PIK3CA | PIK3CA | [NP_006209.2](http://www.ncbi.nlm.nih.gov/sites/entrez?db=gene&cmd=search&term=5290) |
| PIM1 | PIM1 | [NP_002639.1](http://www.ncbi.nlm.nih.gov/sites/entrez?db=gene&cmd=search&term=5292) |
| PIM2 | PIM2 | [NP_006866.2](http://www.ncbi.nlm.nih.gov/sites/entrez?db=gene&cmd=search&term=11040) |
| PIM3 | PIM3 | [NP_001001852.1](http://www.ncbi.nlm.nih.gov/sites/entrez?db=gene&cmd=search&term=415116) |
| PIP5K1A | PIP5K1A | [AAC50911.1](http://www.ncbi.nlm.nih.gov/sites/entrez?db=gene&cmd=search&term=5394) |
| PIP5K2B | PIP4K2B | [NP_003550.1](http://www.ncbi.nlm.nih.gov/sites/entrez?db=gene&cmd=search&term=8396) |
| PKAC-alpha | PRKACA | [NP_002721.1](http://www.ncbi.nlm.nih.gov/sites/entrez?db=gene&cmd=search&term=5566) |
| PKAC-beta | PRKACB | [NP_002722.1](http://www.ncbi.nlm.nih.gov/sites/entrez?db=gene&cmd=search&term=5567) |
| PKMYT1 | PKMYT1 | [NP_872629.1](http://www.ncbi.nlm.nih.gov/sites/entrez?db=gene&cmd=search&term=9088) |
| PKN1 | PKN1 | [NP_998725.1](http://www.ncbi.nlm.nih.gov/sites/entrez?db=gene&cmd=search&term=5585) |
| PKN2 | PKN2 | [NP_006247.1](http://www.ncbi.nlm.nih.gov/sites/entrez?db=gene&cmd=search&term=5586) |
| PLK1 | PLK1 | [NP_005021.2](http://www.ncbi.nlm.nih.gov/sites/entrez?db=gene&cmd=search&term=5347) |
| PLK3 | PLK3 | [NP_004064.2](http://www.ncbi.nlm.nih.gov/sites/entrez?db=gene&cmd=search&term=1263) |
| PLK4 | PLK4 | [NP_055079.2](http://www.ncbi.nlm.nih.gov/sites/entrez?db=gene&cmd=search&term=10733) |
| PRKCD | PRKCD | [NP_006245.2](http://www.ncbi.nlm.nih.gov/sites/entrez?db=gene&cmd=search&term=5580) |
| PRKCE | PRKCE | [NP_005391.1](http://www.ncbi.nlm.nih.gov/sites/entrez?db=gene&cmd=search&term=5581) |
| PRKCH | PRKCH | [NP_006246.2](http://www.ncbi.nlm.nih.gov/sites/entrez?db=gene&cmd=search&term=5583) |
| PRKD1 | PRKD1 | NP_002733.2 |
| PRKCQ | PRKCQ | [NP_006248.1](http://www.ncbi.nlm.nih.gov/sites/entrez?db=gene&cmd=search&term=5588) |
| PRKD2 | PRKD2 | [NP_057541.2](http://www.ncbi.nlm.nih.gov/sites/entrez?db=gene&cmd=search&term=25865) |
| PRKD3 | PRKD3 | [NP_005804.1](http://www.ncbi.nlm.nih.gov/sites/entrez?db=gene&cmd=search&term=23683) |
| PRKG1 | PRKG1 | [NP_006249.1](http://www.ncbi.nlm.nih.gov/sites/entrez?db=gene&cmd=search&term=5592) |
| PRKG2 | PRKG2 | [NP_006250.1](http://www.ncbi.nlm.nih.gov/sites/entrez?db=gene&cmd=search&term=5593) |
| PRKR | EIF2AK2 | [NP_002750.1](http://www.ncbi.nlm.nih.gov/sites/entrez?db=gene&cmd=search&term=5610) |
| PRKX | PRKX | [NP_005035.1](http://www.ncbi.nlm.nih.gov/sites/entrez?db=gene&cmd=search&term=5613) |
| PYK2 | PTK2B | [NP_775267.1](http://www.ncbi.nlm.nih.gov/sites/entrez?db=gene&cmd=search&term=2185) |
| RAF1 | RAF1 | [NP_002871.1](http://www.ncbi.nlm.nih.gov/sites/entrez?db=gene&cmd=search&term=5894) |
| RET | RET | [NP_065681.1](http://www.ncbi.nlm.nih.gov/sites/entrez?db=gene&cmd=search&term=5979) |
| RIOK1 | RIOK1 | [NP_113668.2](http://www.ncbi.nlm.nih.gov/sites/entrez?db=gene&cmd=search&term=83732) |
| RIOK3 | RIOK3 | [NP_003822.2](http://www.ncbi.nlm.nih.gov/sites/entrez?db=gene&cmd=search&term=8780) |
| RIPK1 | RIPK1 | [NP_003795.2](http://www.ncbi.nlm.nih.gov/sites/entrez?db=gene&cmd=search&term=8737) |
| RIPK2 | RIPK2 | [NP_003812.1](http://www.ncbi.nlm.nih.gov/sites/entrez?db=gene&cmd=search&term=8767) |
| ROS1 | ROS1 | [NP_002935.2](http://www.ncbi.nlm.nih.gov/sites/entrez?db=gene&cmd=search&term=6098) |
| RPS6KA1(Kin.Dom.1-N-terminal) | RPS6KA1 | [NP_002944.2](http://www.ncbi.nlm.nih.gov/sites/entrez?db=gene&cmd=search&term=6195) |
| RPS6KA1(Kin.Dom.2-C-terminal) | RPS6KA1 | [NP_002944.2](http://www.ncbi.nlm.nih.gov/sites/entrez?db=gene&cmd=search&term=6195) |
| RPS6KA2(Kin.Dom.1-N-terminal) | RPS6KA2 | [NP_066958.2](http://www.ncbi.nlm.nih.gov/sites/entrez?db=gene&cmd=search&term=6196) |
| RPS6KA2(Kin.Dom.2-C-terminal) | RPS6KA2 | [NP_001006933.1](http://www.ncbi.nlm.nih.gov/sites/entrez?db=gene&cmd=search&term=6196) |
| RPS6KA3(Kin.Dom.1-N-terminal) | RPS6KA3 | [NP_004577.1](http://www.ncbi.nlm.nih.gov/sites/entrez?db=gene&cmd=search&term=6197) |
| RPS6KA4(Kin.Dom.1-N-terminal) | RPS6KA4 | [NP_001006945.1](http://www.ncbi.nlm.nih.gov/sites/entrez?db=gene&cmd=search&term=8986) |
| RPS6KA4(Kin.Dom.2-C-terminal) | RPS6KA4 | [NP_003933.1](http://www.ncbi.nlm.nih.gov/sites/entrez?db=gene&cmd=search&term=8986) |
| RPS6KA5(Kin.Dom.1-N-terminal) | RPS6KA5 | [NP_872198.1](http://www.ncbi.nlm.nih.gov/sites/entrez?db=gene&cmd=search&term=9252) |
| RPS6KA5(Kin.Dom.2-C-terminal) | RPS6KA5 | [NP_004746.2](http://www.ncbi.nlm.nih.gov/sites/entrez?db=gene&cmd=search&term=9252) |
| RPS6KA6(Kin.Dom.1-N-terminal) | RPS6KA6 | [NP_055311.1](http://www.ncbi.nlm.nih.gov/sites/entrez?db=gene&cmd=search&term=27330) |
| RPS6KA6(Kin.Dom.2-C-terminal) | RPS6KA6 | [NP_055311.1](http://www.ncbi.nlm.nih.gov/sites/entrez?db=gene&cmd=search&term=27330) |
| SgK085 | LOC340156 | [NP_001012418.1](http://www.ncbi.nlm.nih.gov/sites/entrez?db=gene&cmd=search&term=340156) |
| SIK | SNF1LK | [NP_775490.2](http://www.ncbi.nlm.nih.gov/sites/entrez?db=gene&cmd=search&term=150094) |
| SIK2 | SNF1LK2 | [NP_056006.1](http://www.ncbi.nlm.nih.gov/sites/entrez?db=gene&cmd=search&term=23235) |
| SLK | SLK | [NP_055535.2](http://www.ncbi.nlm.nih.gov/sites/entrez?db=gene&cmd=search&term=9748) |
| SNARK | NUAK2 | [NP_112214.1](http://www.ncbi.nlm.nih.gov/sites/entrez?db=gene&cmd=search&term=81788) |
| SRC | SRC | [NP_005408.1](http://www.ncbi.nlm.nih.gov/sites/entrez?db=gene&cmd=search&term=6714) |
| SRMS | SRMS | [NP_543013.1](http://www.ncbi.nlm.nih.gov/sites/entrez?db=gene&cmd=search&term=6725) |
| SRPK1 | SRPK1 | [NP_003128.3](http://www.ncbi.nlm.nih.gov/sites/entrez?db=gene&cmd=search&term=6732) |
| SRPK2 | SRPK2 | [AAC05299.1](http://www.ncbi.nlm.nih.gov/sites/entrez?db=gene&cmd=search&term=6733) |
| STK16 | STK16 | [CAA06700.1](http://www.ncbi.nlm.nih.gov/sites/entrez?db=gene&cmd=search&term=8576) |
| STK33 | STK33 | [NP_112168.1](http://www.ncbi.nlm.nih.gov/sites/entrez?db=gene&cmd=search&term=65975) |
| STK36 | STK36 | [NP_056505.1](http://www.ncbi.nlm.nih.gov/sites/entrez?db=gene&cmd=search&term=27148) |
| SYK | SYK | [NP_003168.2](http://www.ncbi.nlm.nih.gov/sites/entrez?db=gene&cmd=search&term=6850) |
| TEC | TEC | [NP_003206.1](http://www.ncbi.nlm.nih.gov/sites/entrez?db=gene&cmd=search&term=7006) |
| TESK1 | TESK1 | [NP_006276.2](http://www.ncbi.nlm.nih.gov/sites/entrez?db=gene&cmd=search&term=7016) |
| TGFBR1 | TGFBR1 | [NP_004603.1](http://www.ncbi.nlm.nih.gov/sites/entrez?db=gene&cmd=search&term=7046) |
| TGFBR2 | TGFBR2 | [NP_003233.4](http://www.ncbi.nlm.nih.gov/sites/entrez?db=gene&cmd=search&term=7048) |
| TIE1 | TIE1 | [NP_005415.1](http://www.ncbi.nlm.nih.gov/sites/entrez?db=gene&cmd=search&term=7075) |
| TIE2 | TEK | [NP_000450.2](http://www.ncbi.nlm.nih.gov/sites/entrez?db=gene&cmd=search&term=7010) |
| TLK1 | TLK1 | [NP_036422.3](http://www.ncbi.nlm.nih.gov/sites/entrez?db=gene&cmd=search&term=9874) |
| TLK2 | TLK2 | [AAF03095.1](http://www.ncbi.nlm.nih.gov/sites/entrez?db=gene&cmd=search&term=11011) |
| TNIK | TNIK | [NP_055843.1](http://www.ncbi.nlm.nih.gov/sites/entrez?db=gene&cmd=search&term=23043) |
| TNK1 | TNK1 | [NP_003976.2](http://www.ncbi.nlm.nih.gov/sites/entrez?db=gene&cmd=search&term=8711) |
| TNK2 | TNK2 | [NP_001010938.1](http://www.ncbi.nlm.nih.gov/sites/entrez?db=gene&cmd=search&term=10188) |
| TNNI3K | TNNI3K | [NP_057062.1](http://www.ncbi.nlm.nih.gov/sites/entrez?db=gene&cmd=search&term=51086) |
| TRKA | NTRK1 | [NP_001012331.1](http://www.ncbi.nlm.nih.gov/sites/entrez?db=gene&cmd=search&term=4914) |
| TRKB | NTRK2 | [NP_006171.2](http://www.ncbi.nlm.nih.gov/sites/entrez?db=gene&cmd=search&term=4915) |
| TRKC | NTRK3 | [AAA75374.1](http://www.ncbi.nlm.nih.gov/sites/entrez?db=gene&cmd=search&term=4916) |
| TSSK1B | TSSK1B | [NP_114417.1](http://www.ncbi.nlm.nih.gov/sites/entrez?db=gene&cmd=search&term=83942) |
| TTK | TTK | [NP_003309.2](http://www.ncbi.nlm.nih.gov/sites/entrez?db=gene&cmd=search&term=7272) |
| TXK | TXK | [NP_003319.2](http://www.ncbi.nlm.nih.gov/sites/entrez?db=gene&cmd=search&term=7294) |
| TYK2(JH2domain-pseudokinase) | TYK2 | [NP_003322.2](http://www.ncbi.nlm.nih.gov/sites/entrez?db=gene&cmd=search&term=7297) |
| TYRO3 | TYRO3 | [NP_006284.2](http://www.ncbi.nlm.nih.gov/sites/entrez?db=gene&cmd=search&term=7301) |
| VEGFR2 | KDR | [NP_002244.1](http://www.ncbi.nlm.nih.gov/sites/entrez?db=gene&cmd=search&term=3791) |
| WEE1 | WEE1 | [NP_003381.1](http://www.ncbi.nlm.nih.gov/sites/entrez?db=gene&cmd=search&term=7465) |
| YANK2 | STK32B | [NP_060871.1](http://www.ncbi.nlm.nih.gov/sites/entrez?db=gene&cmd=search&term=55351) |
| YANK3 | STK32C | [NP_775846.2](http://www.ncbi.nlm.nih.gov/sites/entrez?db=gene&cmd=search&term=282974) |
| YES | YES1 | [NP_005424.1](http://www.ncbi.nlm.nih.gov/sites/entrez?db=gene&cmd=search&term=7525) |
| YSK1 | STK25 | [NP_006365.2](http://www.ncbi.nlm.nih.gov/sites/entrez?db=gene&cmd=search&term=10494) |
| ZAK | ZAK | [NP_598407.1](http://www.ncbi.nlm.nih.gov/sites/entrez?db=gene&cmd=search&term=51776) |
| ZAP70 | ZAP70 | [NP_997402.1](http://www.ncbi.nlm.nih.gov/sites/entrez?db=gene&cmd=search&term=7535) |
