# Supplementary figures and images for: Identification of a Kinase Profile that Predicts Chromosome Damage Induced by Small Molecule Kinase Inhibitors
Source: PLoS Comput Biol. 2009 Jul 24;5(7):e1000446. doi: 10.1371/journal.pcbi.1000446 (PMC2704959; doi:10.1371/journal.pcbi.1000446)

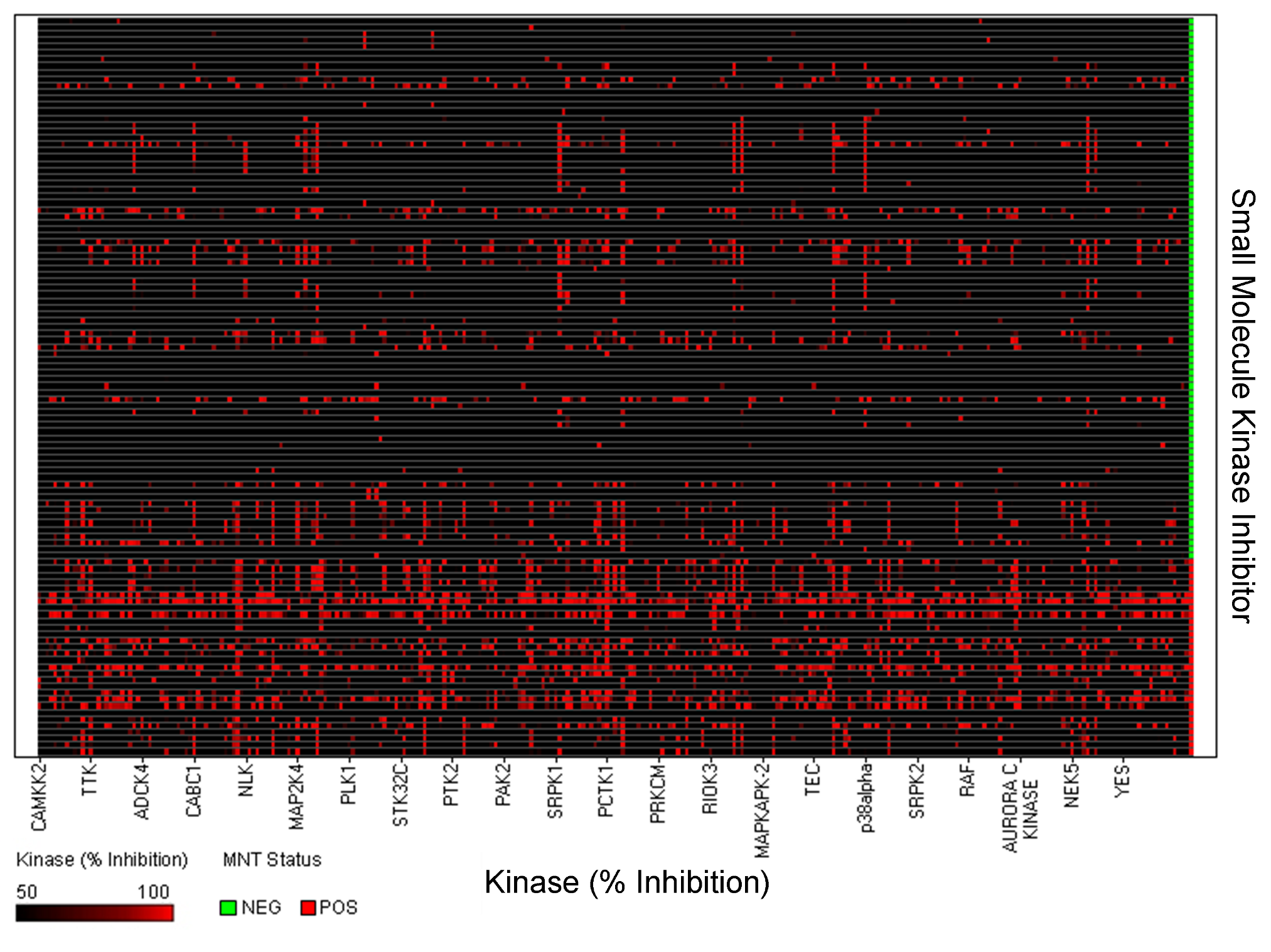

Supplement: Figure S1 — Kinase inhibition heat map of the 113 small molecule kinase inhibitors assayed for micronuclei and the 290 Ambit panel. (0.93 MB TIF) [file pcbi.1000446.s002.tif]
